# Coherent lamellar intergrowth in alkali feldspar

**DOI:** 10.1007/s00410-023-02059-z

**Published:** 2023-10-17

**Authors:** E. Petrishcheva, D. Heuser, R. Abart

**Affiliations:** Department of Lithospheric Research, University of Vienna, 1090 Vienna, Austria

**Keywords:** Alkali feldspar solid solution, Coherent lamellar intergrowth from exsolution, Elastic energy contribution to Gibbs energy, Coherent solvus

## Abstract

A thermodynamic analysis of coherent lamellar intergrowth resulting from the exsolution of initially homogeneous alkali feldspar is presented. In contrast to earlier treatments, where the simplifying assumption of zero strain in the lamellar interfaces was used, our treatment is more general. The elastic stresses and strains associated with coherent lamellar intergrowth of Na-rich and K-rich alkali feldspar are calculated by minimising the overall elastic energy of the lamellar microstructure. At given pressure and temperature, the elastic energy depends on the volume proportions of the two lamellar types, and thus on the composition of the homogeneous precursor feldspar. As a consequence, there is no single coherent solvus for alkali feldspar, but coherent solvi are different for different compositions of the homogeneous precursor phase. Experimentally observed lamellar orientations agree with those predicted by minimising the strain energy on a set of all possible lamellar orientations.

## Introduction

Alkali feldspar is one of the most abundant minerals in the Earth’s crust (Smith and Brown 1988). It forms a solid solution between albite ($${\text{NaAlSi}}_3$$$${\text{O}}_8$$) and K-feldspar ($${\text{KAlSi}}_3$$$${\text{O}}_8$$). Its crystal structure comprises a 3D framework of corner sharing [$${\text{SiO}}_4$$] and [$${\text{AlO}}_4^{-}$$] tetrahedra, and the $${\text{Na}}^{+}$$ and $${\text{K}}^{+}$$ cations occupy relatively large cavities in the Al,Si–O tetrahedral framework (Ribbe 1983). Above about $$550^{\circ}{\text{C}}$$, alkali feldspar with high degree of Si–Al disorder on the tetrahedral sites shows complete miscibility. Towards lower temperatures, a miscibility gap opens. When alkali feldspar of intermediate composition is cooled from super-solvus temperatures into the two-phase region of the phase diagram, it tends to exsolve forming typical lamellar intergrowth of Na-rich and K-rich alkali feldspar, a microstructure referred to as *perthite* (Parsons et al. 2015). Cooling-induced exsolution occurs by the segregation of $${\text{Na}}^{+}$$ and $${\text{K}}^{+}$$ on the extra-framework cation sites, whilst the tetrahedral framework remains unchanged, except for small distortions. As a consequence, the crystal structure is coherent across the lamellar interfaces, a feature that may be preserved over geological times (Abart et al. 2009b; Parsons et al. 2013, 2015). The lattice parameters of alkali feldspar show strong compositional dependence, which is highly anisotropic. The *a*, *b* and *c* lattice parameters increase by about 5.5%, 1.2% and 1.1%, respectively, when the composition changes from pure albite to pure K-feldspar (Kroll et al. 1986; Angel et al. 2012). Chemical segregation within a coherent Al, Si–O tetrahedral framework, thus, causes elastic stresses and strains. The associated elastic energy counteracts chemical segregation, and accordingly, the solvus for coherent intergrowth lies below the so called *strain-free solvus* (Brown and Parsons 1984). The thermodynamics and kinetics of coherent intergrowth resulting from exsolution of an initially homogeneous phase was analysed by Cahn (1961) for isotropic and Cahn (1962) for cubic solids. In Cahn’s thermodynamic formulation, the elastic energy is explicitly accounted for in the Gibbs energy by adding the term (Cahn 1961, 1962)

$$\begin{aligned} {{\mathcal {E}}}_\text {el}= k \left( X - X_0\right) ^2, \end{aligned}$$

where ${{\mathcal {E}}}_\text {el}$ is elastic energy, *X* and $X_0$ are the compositions of one of the segregated phases and of the homogeneous precursor phase, respectively, expressed in terms of the mole fraction of one of the end-member components of the solution phase. The parameter *k* is obtained from calculating the energy associated with the elastic strain that needs to be applied to the segregated phases to make their crystal structures coherent across the interfaces. Cahn’s model was generalised for solids of any crystal symmetry and adopted for application to lamellar coherent intergrowth in alkali feldspar by Willaime and Brown (1974) and Robin (1974). In both studies, the mechanical problem was simplified by requiring that the total strain associated with exsolution is zero in the lamellar interfaces. This is a rather specific scenario, which would be approximated only, when—at a given temperature—the composition of the homogeneous precursor phase lies in the two-phase region of the phase diagram and infinitesimally close to the coherent solvus. In such case, exsolution would produce a volumetrically by far dominating majority phase containing vanishingly thin lamellae of a minority phase. Finite elastic strain would only occur in the minority phase, whilst the majority phase would remain unstrained, so that the total strain at the lamellar interfaces would be zero. For all other compositions of the homogeneous precursor phase that lie within the two-phase region of the phase diagram, the crystal structures of the different lamellae will be strained also in the plane of the lamellar interfaces relative to the crystal structure in the homogeneous precursor phase. This needs to be accounted for in the thermodynamic analysis of coherent intergrowths.

We present calculations of the elastic energy associated with coherent lamellar intergrowth that relax the constraint of zero strain in the plane of lamellar interfaces. Our analysis accounts for the compositional and temperature dependence of the elastic energy. It is shown that the total elastic energy associated with coherent intergrowths and, thus, the coherent solvus depends on the composition of the homogeneous precursor phase.

Last but not least, previous studies on exsolution producing coherent lamellar intergrowth followed a simplified approach. Specifically, one starts from the compositions of the separated phases (binodal points) and just calculates the elastic energy that is required to match the two lattices to each other. However, a new expression for the system-free energy yields new binodal points, and these are not the compositions we started with. To address this issue, we developed an iterative procedure. After calculating the elastic energy, the binodal points are recalculated, and the entire procedure is repeated until the values of the binodal concentrations and the elastic energy no longer change.

Our approach is demonstrated for coherent lamellar intergrowth in alkali feldspar. The method is, however, general and can be applied to any coherent lamellar intergrowth.

## Calculation of the elastic energy

### Problem posing

We first quantify the contribution of the elastic energy to the Gibbs energy of a system consisting of a coherent lamellar intergrowth of Na-rich and K-rich alkali feldspar. Specifically, an initially homogeneous precursor alkali feldspar is assumed to exsolve due to cooling, giving rise to the formation of an alternation of relatively Na-rich and K-rich layers, which are arranged in a sandwich-like lamellar structure. As the lateral extension of each lamella is large compared to its thickness, the lamellar structure is mathematically approximated by a set of closely spaced parallel planar interfaces. The key fact is that the experimentally observed crystal structures are continuous across the interfaces (Tullis and Yund 1979; Yund and Tullis 19683; Evangelakakis et al. 1993; Petrishcheva et al. 2020). The latter are then coherent implying intrinsic stresses.

Although the initial system state is obliterated by exsolution, the lattice of the homogeneous precursor feldspar provides a convenient reference. In what follows, $X_0$ refers to the mole fraction of the K end-member, and ${{\textbf{a}}}$, ${{\textbf{b}}}$, ${{\textbf{c}}}$ refer to the stress-free lattice vectors of the homogeneous precursor alkali feldspar in its *initial state*. As to the mole fraction *X* of the K end-member and the lattice vectors in the Na-rich and K-rich phases produced by exsolution, we use $X_1$, ${{\textbf{a}}}'$, ${{\textbf{b}}}'$, ${{\textbf{c}}}'$ and $X_2$, ${{\textbf{a}}}''$, ${{\textbf{b}}}''$, ${{\textbf{c}}}''$, respectively. The lattice parameters are given as functions of *X* in Kroll et al. (1986). Since during exsolution, ${\text{K}}^{+}$ ions are transported from the Na-rich to the K-rich phase, we have $X_1<X_0<X_2$. The actual lattice parameters of the two phases result from both their compositions and the elastic strains required to maintain coherency between the two phases. The coherency strains $\epsilon '$ and $\epsilon ''$ as well as the induced stresses $\sigma '$ and $\sigma ''$ are of interest.

More precisely, as exsolution develops, the compositions of both phases continuously evolve from the common initial value $X_0$ to the binodal values $X_{b1}$ and $X_{b2}$. This compositional change is accompanied by a change of the lattice parameters, such that chemical strains, denoted by $\upsilon '$ and $\upsilon ''$, are created. In turn, the chemical strains yield the elastic ones, which apply to both the ${{\textbf{a}}}',{{\textbf{b}}}',{{\textbf{c}}}'$ and ${{\textbf{a}}}'',{{\textbf{b}}}'',{{\textbf{c}}}''$ lattices and modify them to maintain coherency of the crystal structures at lamellar interfaces. That is where the elastic energy comes from. We assume that there are no other sources for elastic energy. We follow Robin (1974), who showed (his Appendix A) that under the assumption that the compositionally distinct lamellae are compositionally homogeneous internally, all components of the stress and strain tensors are constant within each lamella.

### Chemical strain

We begin by calculating the stress-free chemical strain for the Na-rich phase relative to the homogeneous precursor phase. The strain components are denoted by $\upsilon '_{ij}$, they depend on the “old” and “new” lattice vectors. In the course of any continuous deformation, a generic position vector ${\textbf{r}}$ of the initial lattice attains a new position ${\textbf{r}}+{\textbf{u}}$ with the same discrete coordinates in the new lattice

$$\begin{aligned} {\textbf{r}} = n_a{{\textbf{a}}}+ n_b{{\textbf{b}}}+ n_c{{\textbf{c}}},\\ {\textbf{r}}+{\textbf{u}} = n_a{{\textbf{a}}}'+ n_b{{\textbf{b}}}'+ n_c{{\textbf{c}}}'. \end{aligned}$$

The displacement vector

$$\begin{aligned} {\textbf{u}} = n_a({{\textbf{a}}}'-{{\textbf{a}}}) + n_b({{\textbf{b}}}'-{{\textbf{b}}}) + n_c({{\textbf{c}}}'-{{\textbf{c}}}), \end{aligned}$$

should be expressed as ${\textbf{u}}({\textbf{r}})$, to comply with the standard definition of the strain tensor. This can be done using the reciprocal basis of the unperturbed lattice ${{\textbf{a}}}^*$, ${{\textbf{b}}}^*$, ${{\textbf{c}}}^*$, which leaves us with

$$\begin{aligned} {\textbf{u}}= ({{\textbf{a}}}^*{\textbf{r}})({{\textbf{a}}}'-{{\textbf{a}}})+ ({{\textbf{b}}}^*\textbf{r})({{\textbf{b}}}'-{{\textbf{b}}})+ ({{\textbf{c}}}^*{\textbf{r}})({{\textbf{c}}}'-{{\textbf{c}}}). \end{aligned}$$

Following the definition $\upsilon _{ij} = \frac{1}{2}(\partial u_i/\partial x_j+\partial u_j/\partial x_i)$, we obtain the chemical strain tensor

1 $$\begin{aligned} \upsilon '_{ij}=\frac{ ({{\textbf{a}}}'-{{\textbf{a}}})_i{{\textbf{a}}}_j^*+ ({{\textbf{b}}}'-{{\textbf{b}}})_i{{\textbf{b}}}_j^*+ ({{\textbf{c}}}'-{{\textbf{c}}})_i{{\textbf{c}}}_j^*}{2} +(i\rightleftarrows j), \end{aligned}$$

for the Na-rich phase. For the K-rich phase, the chemical strain components $\upsilon ''_{ij}$ are calculated in the same way. Recall, that the total strains $\upsilon '+\epsilon '$ and $\upsilon ''+\epsilon ''$ involve additional elastic terms, which are necessary to maintain coherency and contribute to Gibbs energy.

### Monoclinic coordinates

**Fig. 1:**
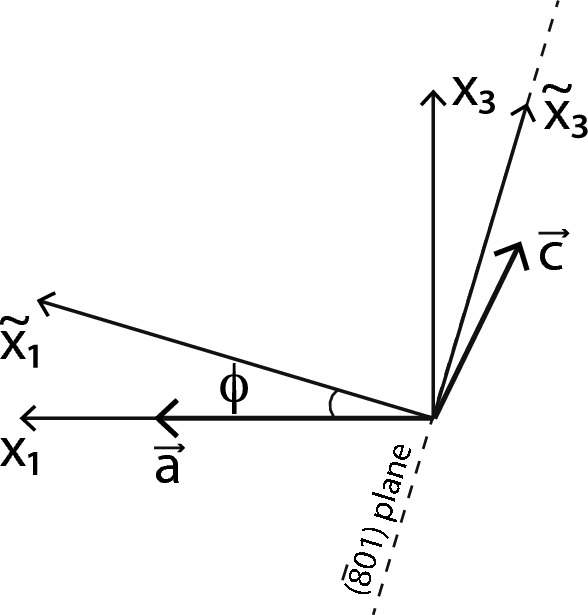
Initial $(x_1,x_3)$ and rotated $(\tilde{x}_1,\tilde{x}_3)$ coordinate systems, where $x_2$ and $\tilde{x}_2$ are identical and $\perp$ to the figure plane. The dashed line shows the trace of the $({\bar{8}}01)$ plane

We assume a monoclinic structure for the initial crystal and for both new phases. Our results, thus, apply above the temperature at which the transition to the triclinic structure occurs. The initial monoclinic crystal cell, in which ${{\textbf{b}}}\perp {{\textbf{a}}},{{\textbf{c}}}$, is fully defined by three lattice parameters *a*, *b*, *c* and one angle $\beta =\widehat{{{\textbf{a}}},{{\textbf{c}}}}$. We choose an orthogonal coordinate system, where $x_1\parallel {{\textbf{a}}}$, $x_2\parallel {{\textbf{b}}}$, and ${{\textbf{c}}}$ belong to the $(x_1,x_3)$ plane (Fig. 1). The corresponding coordinates of the lattice vectors, which we combine into a single matrix, are

$$\begin{aligned} ({{\textbf{a}}},{{\textbf{b}}},{{\textbf{c}}})= \begin{pmatrix} a &{} 0&{} c \cos \beta \\ 0&{} b&{} 0\\ 0 &{}0 &{}c \sin \beta \end{pmatrix}. \end{aligned}$$

This matrix is used for calculating the chemical strains in Eq. (1).

The standard frame $(x_1,x_2,x_3)$ is well-suited for monoclinic symmetry: both, the elastic strain tensor and the stress tensor take a simple form

2 $$\begin{aligned} \epsilon =\begin{pmatrix} \epsilon _{11}&{} 0&{}\epsilon _{13}\\ 0&{} \epsilon _{22}&{} 0\\ \epsilon _{13}&{}0 &{} \epsilon _{33} \end{pmatrix}, \qquad \sigma =\begin{pmatrix} \sigma _{11}&{} 0&{}\sigma _{13}\\ 0&{} \sigma _{22}&{} 0\\ \sigma _{13}&{}0 &{} \sigma _{33} \end{pmatrix}, \end{aligned}$$

because $x_2$ is the diad axis. The pattern of Eq. (2) is preserved after an arbitrary rotation around the diad axis. Recall that for the case at hand, both $\epsilon _{ij}$ and $\sigma _{ij}$ components are uniform in each lamella, see Robin (1974), Appendix A.

An important fact is that the direction of the lattice vector ${{\textbf{b}}}$ does not change during phase separation, such that the $x_2$ axis is exactly the same for all three crystals, i.e. the crystals with compositions $X_0, X_1, X_2$. Later on, we will use the frame $(x_1,x_2,x_3)$ that is defined with reference to the initial lattice for describing the Na- and K-rich phases. The tensors $\epsilon '$, $\sigma '$ and $\epsilon ''$, $\sigma ''$ shall still follow the general pattern of Eq. (2).

In what follows, we will use Hooke’s law: elastic strains and induced stresses are subject to the relation

$$\begin{aligned} \sigma _{ij}= \sum _{p,q=1}^3c_{ijpq}\epsilon _{pq} \quad \text {or using Voigt's notation}\quad \sigma _i=\sum _{j=1}^6c_{ij}\epsilon _j. \end{aligned}$$

For monoclinic symmetry, the stiffness tensor $c_{ijpq}$ in Voigt’s notation and for the coordinate system specified above takes the form (Nye 1957)

3 $$\begin{aligned} C= \begin{pmatrix} c_{11}&{}c_{12}&{}c_{13}&{}0&{}c_{15}&{}0\\ c_{21}&{}c_{22}&{}c_{23}&{}0&{}c_{25}&{}0\\ c_{31}&{}c_{32}&{}c_{33}&{}0&{}c_{35}&{}0\\ 0&{}0&{}0&{}c_{44}&{}0&{}c_{46}\\ c_{51}&{}c_{52}&{}c_{53}&{}0&{}c_{55}&{}0\\ 0&{}0&{}0&{}c_{64}&{}0&{}c_{66} \end{pmatrix}, \quad \text {where}\quad \begin{pmatrix} \sigma _1\\ \sigma _2\\ \sigma _3\\ 0\\ \sigma _5\\ 0 \end{pmatrix} =C \begin{pmatrix} \epsilon _1\\ \epsilon _2\\ \epsilon _3\\ 0\\ \epsilon _5\\ 0 \end{pmatrix}, \end{aligned}$$

cf. Equation (2). All zeros retain their positions after an arbitrary rotation around the diad axis. Note that Eq. (3) provides four relations. Note also that by construction $\sigma _5=\sigma _{13}$, but $\epsilon _5=2\epsilon _{13}$, see Nye (1957). According to Waeselmann et al. (2016), the stiffness coefficients show only weak compositional dependence, which we disregard in our analysis.

Hence, we use *C* over the entire studied compositional range instead of discerning between *C*, $C'$ and $C''$. The values $c_{ij}$ were taken from Haussuehl (1993). Note that the stiffness coefficients tensor in Haussuehl (1993) is given in the frame where $x_1 \parallel {\textbf{a}}^{\star }$, $x_2 \parallel {\textbf{b}}$ (diad axis) and $x_3 \parallel {\textbf{c}}$. The coefficients are transformed to our coordinates as specified in Table 1.

**Table 1:** Stiffness coefficients in [GPa] for $x_1 \parallel {\textbf{a}}$, $x_2 \parallel {\textbf{b}}$ (diad axis), $x_3 \parallel {\textbf{c}}^{\star }$, taken from Haussuehl (1993)

	$c_{i,1}$	$c_{i,2}$	$c_{i,3}$	$c_{i,4}$	$c_{i,5}$	$c_{i,6}$
$c_{1,j}$	93.9	41.5	52.2	0	$-$26.2	0
$c_{2,j}$	41.5	176.8	23.1	0	14.2	0
$c_{3,j}$	52.2	23.1	82.1	0	$-$19.5	0
$c_{4,j}$	0	0	0	17.8	0	9.7
$c_{5,j}$	$-$26.2	14.2	$-$19.5	0	44.2	0
$c_{6,j}$	0	0	0	9.7	0	35.0

### Rotation

Next, we introduce a rotated coordinate frame that is better adjusted to the lamellar interfaces. The change from $(x_1,x_2,x_3)$ to the new frame $(\tilde{x}_1,\tilde{x}_2,\tilde{x}_3)$ follows general transformation rules of tensor algebra (Nye 1957),

4 $$\begin{aligned} \tilde{\upsilon }_{ij}= & {} \sum _{\alpha ,\beta =1}^3 A_{i\alpha }A_{j\beta }\upsilon _{\alpha \beta }, \quad \tilde{\epsilon }_{ij}=\sum _{\alpha ,\beta =1}^3 A_{i\alpha }A_{j\beta }\epsilon _{\alpha \beta }, \end{aligned}$$

5 $$\begin{aligned} \tilde{c}_{ijkl}= & {} \sum _{\alpha ,\beta ,\gamma ,\delta =1}^3 A_{i\alpha }A_{j\beta }A_{k\gamma }A_{l\delta } c_{\alpha \beta \gamma \delta }, \end{aligned}$$

where the rotation matrix *A* is defined such that $\tilde{x}_i=\sum _{\alpha =1}^3A_{i\alpha }x_\alpha$.

To take advantage of the rotated frame, we need to discuss the spatial arrangement of the interfaces. It is an important experimental fact that the $x_2$ axis is parallel to the lamellar interfaces. We rotate the coordinates $(x_1,x_2,x_3)$ of the homogeneous feldspar around $x_2$, until the new $\tilde{x}_1$ axis becomes orthogonal to the lamellar interfaces, which are then parallel to the $(\tilde{x}_2,\tilde{x}_3)$ plane (see Fig. 1). Experimentally observed lamellar interfaces have orientations corresponding to lattice planes with Miller indices in the range of $(\bar{6} 0 1)$ to $(\bar{8} 0 1)$. One can then calculate the required rotation angle $\phi$ and the corresponding rotation matrix. For lamellar orientations containing the ${{\textbf{b}}}$ axis, we obtain

6 $$\begin{aligned} A= \begin{pmatrix} \cos \phi &{} 0&{} \sin \phi \\ 0&{} 1&{} 0\\ -\sin \phi &{}0 &{}\cos \phi \end{pmatrix}, \end{aligned}$$

where $\phi$ refers to the lattice parameters of the homogeneous precursor feldspar. In our calculations, we use a lamellar orientation of $(\bar{8} 0 1)$, for which the exact value of $\phi$ varies between $17.8^\circ$ and $18.6^\circ$, depending on the composition $X_0$ of the feldspar. Matrix *A* will be used with Eqs. (4, 5). Note that the rotation is around the diad axis and preserves the general structure of the second-order tensors like the ones in Eq. (2) and of the fourth-order tensor like the one in Eq. (3).

In summary, we describe coherent lamellar intergrowth in a rotated frame of reference in the following way:

- Chemical strains are first calculated in the coordinate system referring to the homogeneous feldspar using Eq. (1) and then transformed to the rotated coordinates using Eq. (4) with matrix (6). The procedure provides $\tilde{\upsilon }'$ and $\tilde{\upsilon }''$.
- All stress and strain tensors follow the pattern of Eq. (2) both before and after rotation. In the rotated coordinates, one can prove that (Robin 1974) $\tilde{\sigma }_{11}'=\tilde{\sigma }_{13}'=0$ and $\tilde{\sigma }_{11}''=\tilde{\sigma }_{13}''=0$.
- In the rotated coordinates, Hooke’s law takes the form
  7 $$\begin{aligned} \begin{pmatrix} 0\\ \tilde{\sigma }_2'\\ \tilde{\sigma }_3'\\ 0\\ 0\\ 0 \end{pmatrix} =\tilde{C} \begin{pmatrix} \tilde{\epsilon }_1'\\ \tilde{\epsilon }_2'\\ \tilde{\epsilon }_3'\\ 0\\ \tilde{\epsilon }_5'\\ 0 \end{pmatrix}, \quad \begin{pmatrix} 0\\ \tilde{\sigma }_2''\\ \tilde{\sigma }_3''\\ 0\\ 0\\ 0 \end{pmatrix} =\tilde{C} \begin{pmatrix} \tilde{\epsilon }_1''\\ \tilde{\epsilon }_2''\\ \tilde{\epsilon }_3''\\ 0\\ \tilde{\epsilon }_5''\\ 0 \end{pmatrix}, \end{aligned}$$
  where $\tilde{C}$ is the stiffness tensor *C* in the rotated coordinate system. Here $\tilde{C}$ follows the pattern of Eq. (3). Actually, $\tilde{C}$ is used instead of $\tilde{C}'$ and $\tilde{C}''$ for the reasons given above.
- Coherency at the lamellar interfaces, which are parallel to the $(\tilde{x}_2,\tilde{x}_3)$ plane, requires that the total strain $\tilde{\upsilon }+\tilde{\epsilon }$ remains continuous, i.e.
  $$\begin{aligned} (\tilde{\upsilon }'+\tilde{\epsilon }')_{ij} = (\tilde{\upsilon }''+\tilde{\epsilon }'')_{ij} \quad \text {for}\quad i,j=2,3, \end{aligned}$$
  which for the case at hand yields two relations
  8 $$\begin{aligned} \tilde{\upsilon }_2'+\tilde{\epsilon }_2'= \tilde{\upsilon }_2''+\tilde{\epsilon }_2'', \quad \text {and}\quad \tilde{\upsilon }_3'+\tilde{\epsilon }_3'= \tilde{\upsilon }_3''+\tilde{\epsilon }_3''. \end{aligned}$$

Note that each matrix equation in (7) provides four nontrivial relations, cf. Equation (3). Altogether, Eqs. (7, 8) provide 10 relations for 12 unknowns: 4 stress and 8 elastic strain components. In the following, we solve the underdetermined problem by minimising the elastic energy.

### Energy minimisation

The volume density of the elastic energy accumulated in the Na- and the K-rich phases due to stresses is given by the standard relations (Nye 1957)

9 $$\begin{aligned} {{\mathcal {E}}}_\text {el}'= \frac{1}{2}\sum _{i=1}^6\sigma '_i\epsilon '_i, \qquad {{\mathcal {E}}}_\text {el}''= \frac{1}{2}\sum _{i=1}^6\sigma ''_i\epsilon ''_i. \end{aligned}$$

The resulting elastic energy per unit volume of lamellar intergrowth that comprise many layers is given by

$$\begin{aligned} {{\mathcal {E}}}_\text {el}= \omega {{\mathcal {E}}}_\text {el}'+ (1-\omega ){{\mathcal {E}}}_\text {el}'', \end{aligned}$$

where $\omega$ and $1-\omega$ are the volume fractions occupied by the Na-rich and the K-rich lamellae. Recall that $X_1<X_0<X_2$ are the K end-member mole fractions of the Na-rich, initial and K-rich phases, respectively. For calculating the volume fractions, we neglect the compositional dependence of the molar volume such that

10 $$\begin{aligned} X_0= \omega X_1+ (1-\omega )X_2\quad \Rightarrow \quad \omega = \frac{X_2-X_0}{X_2-X_1}. \end{aligned}$$

The energies in Eq. (9) have the same values in all coordinate systems. Calculations are most conveniently done in the rotated frame, because most stresses vanish. Using Eq. (7), we obtain

11 $$\begin{aligned} {{\mathcal {E}}}_\text {el}= & {} \frac{1}{2}\frac{X_2-X_0}{X_2-X_1}\sum _{i=2,3}( \tilde{c}_{i1}\tilde{\epsilon }_1'+ \tilde{c}_{i2}\tilde{\epsilon }_2'+ \tilde{c}_{i3}\tilde{\epsilon }_3'+ \tilde{c}_{i5}\tilde{\epsilon }_5' )\tilde{\epsilon }_i' \nonumber \\{} & {} \quad +\frac{1}{2}\frac{X_0-X_1}{X_2-X_1}\sum _{i=2,3}( \tilde{c}_{i1}\tilde{\epsilon }_1''+ \tilde{c}_{i2}\tilde{\epsilon }_2''+ \tilde{c}_{i3}\tilde{\epsilon }_3''+ \tilde{c}_{i5}\tilde{\epsilon }_5'' )\tilde{\epsilon }_i''. \end{aligned}$$

Here, the elastic strain components should be chosen such that ${{\mathcal {E}}}_\text {el}$ is minimised.

From the mathematical point of view, ${{\mathcal {E}}}_\text {el}$ is a positive-defined quadratic form (Strang 2005). This form should be minimised with respect to eight involved strain variables, whilst at the same time, accounting for the six linear constraints

$$\begin{aligned} \tilde{c}_{11}\tilde{\epsilon }_1'+ \tilde{c}_{12}\tilde{\epsilon }_2'+ \tilde{c}_{13}\tilde{\epsilon }_3'+ \tilde{c}_{15}\tilde{\epsilon }_5'&= 0, \\ \tilde{c}_{51}\tilde{\epsilon }_1'+ \tilde{c}_{52}\tilde{\epsilon }_2'+ \tilde{c}_{53}\tilde{\epsilon }_3'+ \tilde{c}_{55}\tilde{\epsilon }_5'&= 0,&\tilde{\epsilon }_2' -\tilde{\epsilon }_2''&= \tilde{\upsilon }_2''-\tilde{\upsilon }_2', \\ \tilde{c}_{11}\tilde{\epsilon }_1''+ \tilde{c}_{12}\tilde{\epsilon }_2''+ \tilde{c}_{13}\tilde{\epsilon }_3''+ \tilde{c}_{15}\tilde{\epsilon }_5''&= 0,&\tilde{\epsilon }_3' -\tilde{\epsilon }_3''&= \tilde{\upsilon }_3''-\tilde{\upsilon }_3', \\ \tilde{c}_{51}\tilde{\epsilon }_1''+ \tilde{c}_{52}\tilde{\epsilon }_2''+ \tilde{c}_{53}\tilde{\epsilon }_3''+ \tilde{c}_{55}\tilde{\epsilon }_5''&= 0, \end{aligned}$$

resulting from Hooke’s law (7) and from the coherency conditions (8).

One can employ the above constraints to eliminate six from the total of eight strain components in Eq. (11) and then minimise ${{\mathcal {E}}}_\text {el}$ manually. Alternatively, a solver can be employed to perform the constrained minimisation. There is always a minimum, as ${{\mathcal {E}}}_\text {el}\ge 0$. It is important to note that, in general, the right-hand side of the last two constraints is non-zero. Minimisation then yields a nontrivial solution: at least some of the elastic strains are non-zero. The above described procedure provides the elastic energy of any lamellar intergrowth of Na-rich and K-rich alkali feldspar provided that information on the end-member mole fractions, chemical strains and stiffness tensor is available. Needless to say that as long as the interfaces contain the diad axis, their orientation may differ from $(\bar{8} 0 1)$, in which case $\phi$ in Eq. (6) would have to be recalculated.

### Gibbs energy

Let us now discuss how a feldspar exsolves during cooling from a thermodynamic point of view. We denote the Gibbs energy density of a homogeneous feldspar under given pressure and temperature as $n_0g(T,P,X)$, where $n_0$ gives the number of moles per unit volume, and *g* is the molar Gibbs energy, which depends on temperature, pressure and composition.

**Fig. 2:**
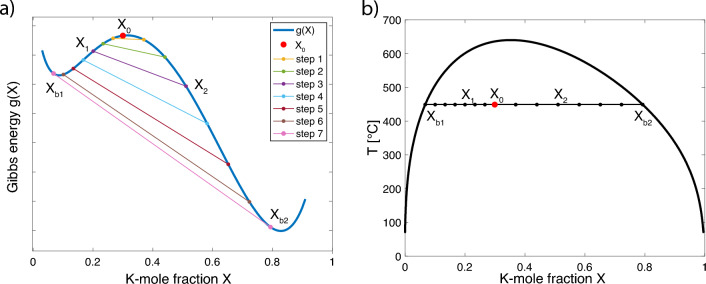
**a** Molar Gibbs energy diagram showing a double-well energy *g* versus composition *X* curve for strain-free akali feldspar and an exemplary exsolution path from the initial composition $X=X_0=0.3$ to the binodal values for $T=450\,^{\circ}{\text{C}}$, $P = 1$ bar. The elastic energies ${{\mathcal {E}}}_\text {el}'(X_1)$ and ${{\mathcal {E}}}_\text {el}''(X_2)$ are permanently evaluated along the path and approximated by $k(X_{1,2}-X_0)^2$, which yields $k(T,X_0)$. **b** Representation of the same path in *X*, *T* coordinates

When dealing with phase transitions, it is not sufficient to know *g* for the initial composition $X=X_0$, also the shape of *g* versus *X* is important. When temperature drops below the critical value, *g*(*X*) changes its shape from being convex towards low values of *g* over the entire compositional range to a double-well shape such as shown in Fig. 2(left). The equilibrium compositions of the Na-rich and the K-rich phase are given by the binodal points $X_{b1}(T,P)$ and $X_{b2}(T,P)$, which are obtained by the common tangent construction (Porter et al. 2021).

A generic expression for the molar Gibbs energy of a binary solution phase is

12 $$\begin{aligned} g(T,P,X)=RT\Bigl (X\ln X+(1-X)\ln (1-X)\Bigr )+g^\text {ex}(T,P,X), \end{aligned}$$

where the first term represents the configurational entropy contribution to Gibbs energy, and $g^\text {ex}$ accounts for the thermodynamic non-ideality of the solution phase. The mechanical-mixture term, which does not affect phase separation, is omitted for brevity. We follow Hovis et al. (1991) and use

13 $$\begin{aligned} g^\text {ex} = X(1-X) \Bigl [X\,W_\text {ab}(T,P) + (1-X)W_\text {or}(T,P)\Bigr ], \end{aligned}$$

where the (Margules) parameters $W_{\text {ab},\text {or}}$ are (Hovis et al. 1991)

$$\begin{aligned} W_\text {ab}/\text {(J/mol)}&=22820-6.3(T/\text{K})+0.461(P/\text {bar}),\\ W_\text {or}/\text {(J/mol)}&=19550-10.5(T/\text{K})+0.327(P/\text {bar}). \end{aligned}$$

For a perthite with coherent interfaces ${{\mathcal {E}}}_\text {el}(T,P,X_0,X)$ arising from the elastic strains and stresses needs to be added to $n_0g(T,P,X)$. To begin with, the elastic energy vanishes for $X=X_0$ and is positive if $X\ne X_0$. It is naturally approximated by

14 $$\begin{aligned} {{\mathcal {E}}}_\text {el}=k(X-X_0)^2, \end{aligned}$$

where $k=k(T,P,X_0)$. For the elastic energies from the previous section, one should set $X=X_1,X_2$ for the Na-rich and the K-rich phases, respectively, such that

$$\begin{aligned} {{\mathcal {E}}}_\text {el}'=k(X_1-X_0)^2, \quad {{\mathcal {E}}}_\text {el}''=k(X_2-X_0)^2=\frac{\omega ^2}{(1-\omega )^2} k(X_1-X_0)^2, \end{aligned}$$

where use has been made of the fact that $X_1$ and $X_2$ are related by Eq. (10). The value of $\omega$ is derived from the binodal compositions, such that we get

15 $$\begin{aligned} {{\mathcal {E}}}_\text {el}=\omega {{\mathcal {E}}}_\text {el}'+ (1-\omega ){{\mathcal {E}}}_\text {el}''=\frac{\omega }{1-\omega }k(X_1-X_0)^2, \end{aligned}$$

with

16 $$\begin{aligned} \omega = \frac{X_{b2}-X_0}{X_{b2}-X_{b1}} \end{aligned}$$

for the total elastic energy.

There are two different reasons why *k* depends on temperature and pressure. First, both lattice parameters and stiffness coefficients change with *P* and *T*. This change is supposed to be small and is disregarded in our analysis. Second, for a given $X_0$, *k* implicitly depends on *T* and *P* via the volume proportions of the Na-rich and the K-rich lamellae, which, in turn, are determined by the binodal points as obtained from *g*(*T*, *P*, *X*). In what follows, *P* takes one fixed value, $P=1\,\text {bar}$ in the example at hand, and only the implicit compositional and temperature dependence of *k* is taken into account.

To find *k* for a given temperature, we consider a set of possible initial compositions $X_0$ in the two-phase region of the phase diagram. We then trace exsolution for each initial $X_0$ from the set. To this end, we gradually change the Na- and K-rich compositions $X_1$ and $X_2$, constrained by Eq. (10), starting from the chosen $X_0$ and ending at the binodal compositions $X_{b1,b2}$ (see Fig. 2). We follow the changing compositions and calculate the elastic energy ${{\mathcal {E}}}_\text {el}$ as a function of $X_1-X_0$ (see Fig. 4). The value of *k* is chosen such that Eq. (15) provides the best approximation for ${{\mathcal {E}}}_\text {el}$. We perform this calculation for each initial $X_0$ and get $k(X_0)$ for a chosen *T*. This procedure is repeated for all temperatures of interest yielding $k(T,X_0)$.

**Fig. 3:**
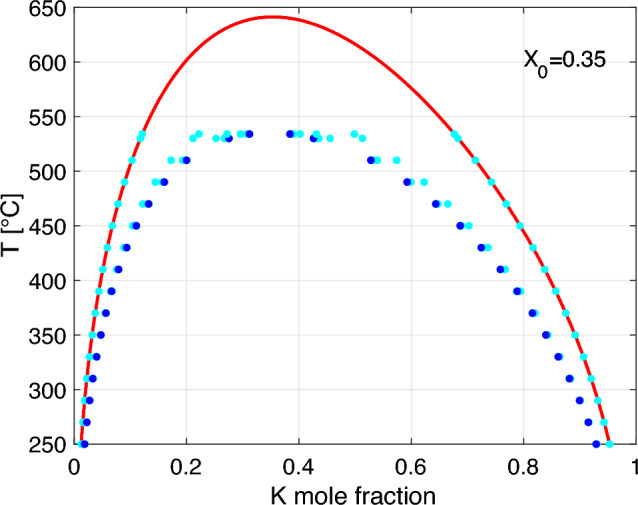
Successive approximations to the binodal compositions for coherent lamellar intergrowth ($X_0=0.35$, $P = 1$ bar). The initial (stress-free) binodal points lie on the strain free solvus (red curve). Each iterative calculation of *k* is followed by the update of the Gibbs energy function and shifts the binodal points (light-blue) parallel to the *x*-axis. Their limiting final positions (dark blue) correctly account for stresses. Our results obtained for different values of $X_0$ are summarised in Fig. 8

It is important to note, that the binodal compositions $X_{b1,b2}$ used for calculating the volume fraction $\omega$ (Eq. 16) are determined by the strain-free *g*(*T*, *P*, *X*). From the procedure described above, we obtain *k*, from which a new *g*(*T*, *P*, *X*), now accounting for the strain energy, is calculated. In general, the new corresponding binodal points yield a new $\omega$. This renders *k*, which was calculated with the previous, different $\omega$, obsolete. To proceed, we repeat the calculation with the new $\omega$ to obtain a better approximation. This procedure converges yielding the correct *k* after a few iterations. Figure 3 shows the successive approximations of the corresponding binodal points.

**Fig. 4:**
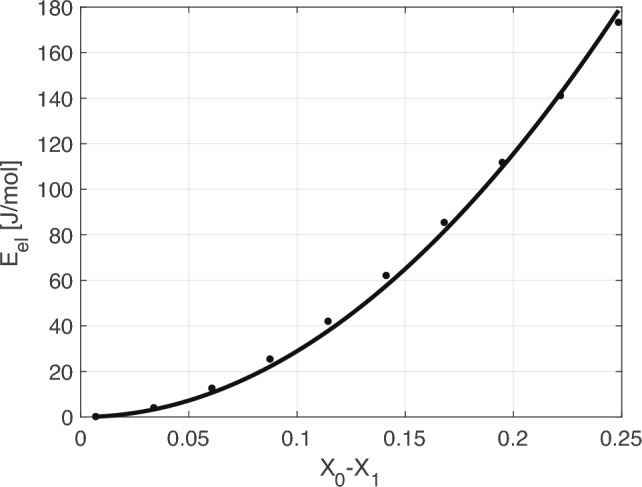
Elastic energy versus $X_0-X_1$ for $X_0=0.35$ and $T=440^\circ$ C, $P = 1$ bar. The numerical calculations are represented by points, the solid line shows predictions of the model equation (15) with a properly fitted *k*. We have performed many such calculation to obtain $k(T,X_0)$

**Fig. 5:**
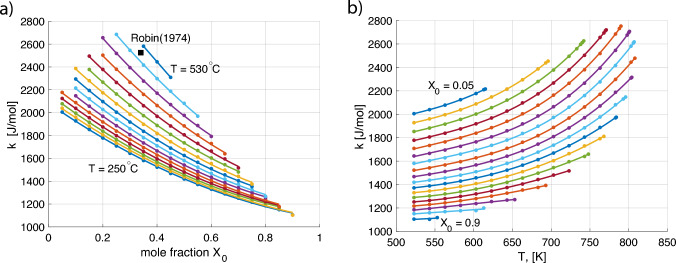
**a** *k* from Eq. (14) versus $X_0$. Each solid line corresponds to a fixed temperature in the range from $250^\circ \text {C}$ to $530^\circ \text {C}$ with $20^\circ \text {C}$ steps. The black square shows *k* after Robin (1974) for comparison. **b** *k* from Eq. (14) versus temperature. Each solid line corresponds to a fixed $X_0$ within the two-phase region of the phase diagram with steps of 0.05. Each point is obtained from a separate iterative calculation of the elastic energy such as shown in Fig. 4. All calculations were done for a pressure of $P = 1$ bar

Note that Eq. (15) provides elastic energy per unit volume. It is multiplied by the molar volume of feldspar (Holland and Powell 2011), $V_\text {mol}=108.4\times 10^{-6}\, \text {m}^3/\text {mol}$, when added to Eq. (13). The resulting dependence of *k* on $X_0$ at a range of fixed temperatures is shown in Fig. 5a. The different curves only cover the compositional range, where $X_0$ falls into the two phase region of the phase diagram, and thus become shorter with increasing temperature. The value of *k* presented by Robin (1974) is shown for comparison. The temperature dependence of *k* for different values of $X_0$ is illustrated in Fig. 5b. Now the curves only cover the temperature range corresponding to the two-phase region of the phase diagram at the respective $X_0$.

## Discussion

### Lamellar orientation

**Fig. 6:**
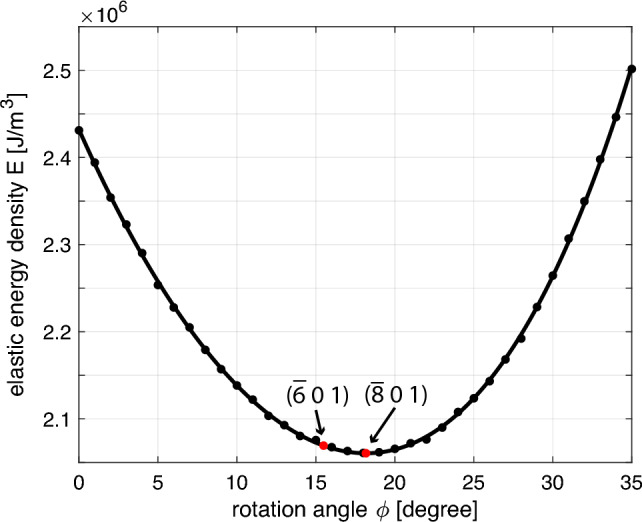
Elastic energy density ${{\mathcal {E}}}_\text {el}$ in [${\text{J}}/{\text{m}}^3$] for coherent lamellar intergrowth versus angle $\phi$, for $T = 400^\circ$C, $P = 1$ bar and $X_0= 0.45$, ${{\mathcal {E}}}_\text {el}$ is minimised at $\phi = 18.2^\circ$ corresponding to Miller indices $(\bar{8}01)$. Red points indicate the range of orientations for experimentally observed lamellar interfaces, which is given by Miller indices ranging from $(\bar{6} 0 1)$ to $(\bar{8} 0 1)$

Exsolution of alkali feldspar may occur via spinodal decomposition or by nucleation and growth (Abart et al. 2009a, b; Petrishcheva and Abart 2009, 2012). Irrespective of the exsolution pathway, the process proceeds towards a configuration ensuring minimum Gibbs energy. In general, the elastic energy associated with coherent lamellar intergrowth varies with lamellar orientation. Naturally, the lamellar orientation is expected to be selected so as to minimise the elastic energy (Willaime and Brown 1974). We calculated the elastic energy ${{\mathcal {E}}}_\text {el}$ for different interface orientations all containing the crystallographic **b** direction. Let the interface orientation be given by $\phi$, the angle enclosed by the crystallographic **a** direction and the normal to the plane of lamellar interfaces (see Fig. 1). Figure 6 shows ${{\mathcal {E}}}_\text {el}$ as a function of $\phi$ and reveals a pronounced orientation-dependent variation of ${{\mathcal {E}}}_\text {el}$ with a clear minimum in the angular range of $0^\circ \le \phi \le 35^\circ$. The minimum of ${{\mathcal {E}}}_\text {el}(\phi )$ is at $\phi = 18.2^\circ$ corresponding to a lamellar orientation parallel to a plane with Miller indices $(\bar{8}01)$, which is well within the range of commonly observed lamellar orientations (Willaime and Brown 1974). It must be noted, however, that similar lamellar orientations were also obtained by Bollmann and Nissen (1968) based on O-lattice theory. Thus, it cannot be decided, to what extent the degree of geometrical match between the two lattices at lamellar interfaces as described by the O-lattice theory, or minimisation of elastic energy control the selection of lamellar orientation. In any case, the orientations of exsolution lamellae in perthites are so that the elastic energy is minimised.

### Thermodynamics of coherent intergrowth

**Fig. 7:**
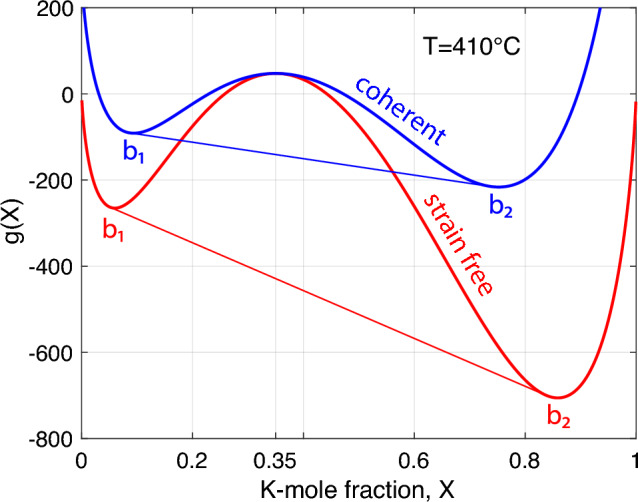
Molar Gibbs energy diagram showing the Gibbs energy for strain-free alkali feldspar (red) calculated from the thermodynamic mixing model of Hovis et al. (1991) (Eq. 12) and for coherent lamellar intergrowth (blue) obtained by adding ${{\mathcal {E}}}_\text {el}$ (Eq. 14) calculated using the elastic constants given in Table 1 to the *g*(*T*, *X*) curve for the strain-free phase relations; $T = 410^\circ$C, $P = 1$ bar and $X_0= 0.35$

Having the elastic energy at our disposal, we can recalculate the Gibbs energy of coherent lamellar intergrowth. In Fig. 7, the Gibbs energy of strain-free alkali feldspar is compared to the Gibbs energy of coherent lamellar intergrowth. The two curves coincide at $X = X_0$, where the chemical and elastic strain and, thus, the elastic energy vanish. The effect of adding the elastic energy is to make the *g*-*X* curve more strongly convex towards low values of *g*, which reflects the fact that the elastic stresses and strains induced by the segregation of ${\text{Na}}^+$ and ${\text{K}}^+$ in a coherent Al,Si–O tetrahedral framework counteract chemical segregation and suppress exsolution. At a given composition $X_0$ of the homogeneous precursor feldspar, exsolution producing coherent intergrowth, thus, occurs at a lower temperature than would be expected from the *g*-*X* curve for strain-free alkali feldspar.

**Fig. 8:**
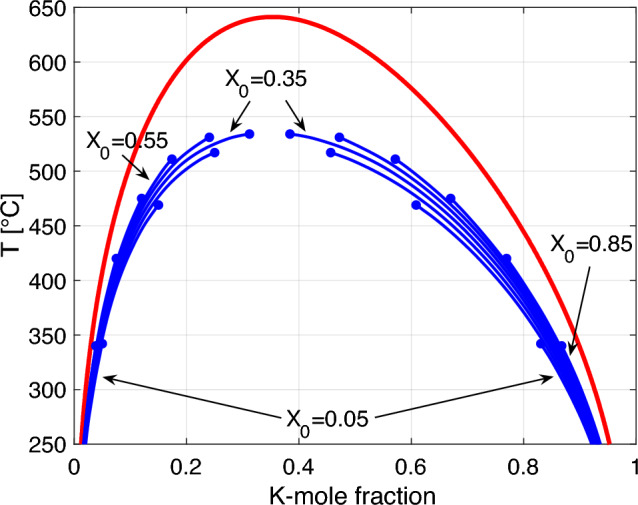
Isobaric *T*-*X* diagram at $P = 1$ bar showing the strain-free solvus (red) as calculated from the thermodynamic mixing model of Hovis et al. (1991) and coherent solvi (blue) for $X_0$ in the range from 0.05 to 0.85 with 0.1 steps obtained by adding ${{\mathcal {E}}}_\text {el}$ (Eq. 14) calculated using the elastic constants given in Table 1 to the *g*(*T*, *X*) curve for the strain-free phase relations

The coherent solvi calculated by Gibbs energy minimisation (Connolly 1990) for different values of $X_0$ lie below the strain-free solvus (Fig. 8). It is important to note that the positions and the exact shapes of the coherent solvi depend on the composition of the homogeneous precursor feldspar. The different solvus curves only extend over the temperature ranges below which the respective $X_0$ lies within the two-phase field of the phase diagram. For the most complete solvus curve, which occurs at $X_0= 0.35$, the critical temperature is about $100 ^\circ$C lower than for strain-free phase equilibria.

Our analysis differs from those of Robin (1974) and of Willaime and Brown (1974), where the coherent solvus does not depend on $X_0$. In these earlier analyses, the parameter *k* was treated as independent of $X_0$. This is due to the fact that both Robin (1974) as well as Willaime and Brown (1974) required that the total strain in the plane of the lamellar interfaces is zero during the transformation of the homogeneous precursor feldspar into a coherent lamellar intergrowth of Na-rich and K-rich alkali feldspar. Accordingly, the left- and right-hand sides of both equations in (8) vanish. In that case, Eq. (8) yields four equations instead of two, and sufficient constraints are available for solving the elastic problem. The presumption of zero strain in the plane of the lamellar interfaces, however, only holds for the case, where the bulk composition of the precursor phase nearly coincides with one of the binodal compositions at the respective temperature, so that vanishingly thin lamellae of the minority phase are embedded in the volumetrically by far dominant majority phase. This is the case that was analysed by Robin (1974). For any bulk composition that is between the binodal points, both lamellar types have finite thickness, and the presumption of zero strain in the plane of lamellar interfaces does not apply. This is why we relax this constraint and solve the undetermined problem of Eq. (11) by minimising the elastic energy.

In our treatment, the fundamental difficulty arises that for calculating $g(T,P,X,X_0)$, the volume proportions of the Na-rich and K-rich phases must be known, which, in turn, requires $g(T,P,X,X_0)$, calling for an iterative method. This was not considered by Robin (1974) and Willaime and Brown (1974). The treatments of these latter authors correspond to the first approximations shown in Fig. 3. The discrepancy between the first approximations and the binodal points that correctly account for stresses (dark blue points in Fig. 3) is large at high temperatures and becomes minute towards low temperatures. Thus, for low temperatures, the treatments of Robin (1974) and Willaime and Brown (1974) yield results that deviate only slightly from the coherent solvus but they deliver unsatisfactory results at high temperatures (see Fig. 3).

**Fig. 9:**
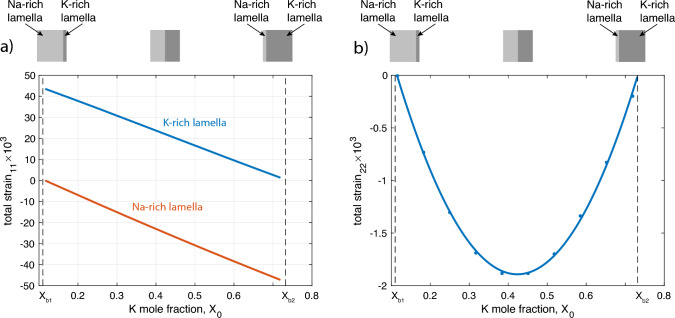
**a** Variation of $\epsilon _{11}$, total strain perpendicular to lamellar interfaces, with $X_0$ at $T = 440^{\circ }\text {C}$ and $P = 1$ bar, $X_{b1}$ and $X_{b2}$ correspond to the binodal compositions. When $X_0$ approaches $X_{b1}$, this corresponds to a configuration with thin lamellae of the K-rich phase in a matrix of the Na-rich phase, and the opposite relations hold, when $X_0$ approaches $X_{b2}$. **b** Variation of $\epsilon _{22}$, total strain parallel to *b*, which is parallel to the lamellar interfaces, with $X_0$

Even if the stiffness coefficients are assumed to be independent of composition and temperature, it is found from our treatment that the total strain in the plane of the lamellar interfaces varies with the volume proportion of the Na-rich and the K-rich lamellae, and thus with $X_0$. The dependence of $\epsilon _{11}$, that is the total strain in the direction perpendicular to the lamellar interfaces, and of $\epsilon _{22}$, the total strain in the direction of the *b* axis, on $X_0$ is shown in Fig. 9. When $X_0$ approaches one of the binodal compositions, the total strain of the majority phase goes to zero (see Fig. 9). For intermediate compositions, the condition that the total strain in the plane of the lamellar interfaces is similar in both the Na-rich and the K-rich phase (Eq. 8) requires distortion of the lattices of both phases, both $\epsilon _{11}$ and $\epsilon _{22}$ assume finite values, to accommodate the difference in molar volume between the Na-rich and the K-rich phase. When $X_0$ approaches one of the binodal compositions, $\epsilon _{11}$ of the majority phase approaches zero and the minority phase experiences maximum shortening/dilation in the direction perpendicular to the lamellar interfaces, whilst $\epsilon _{22}$ goes to zero (see Fig. 9).

Recall, that the compositionally distinct lamellae were considered as homogeneously strained, a scenario that arises, when the different lamellae have uniform compositions internally (Robin 1974). This condition may not be completely fulfilled during the incipient stages of exsolution, when individual lamellae are less than about 20 nm wide and compositional gradients at lamellar interface dominate (Petrishcheva et al. 2020). After coarsening to several 10 s of nanometers, compositional plateaus tend to develop in the lamellae, which are still coherent. In such case, our analysis closely corresponds to the actual configuration. A more sophisticated analysis would be required for configurations, where compositional gradients at the lamellar interfaces dominate.

## Implications

To the best of the authors knowledge, dropping the assumption of zero strain in the plane of lamellar interfaces in the thermodynamic treatment of coherent lamellar intergrowth is novel in mineralogy. It requires solving the mechanical problem by minimising elastic energy. An important implication of this approach is that the equilibrium phase relations in coherent lamellar intergrowth from exsolution depend on the composition of the homogeneous precursor phase. Such an inference was already made by Cahn (1962) for the case where the elastic constants depend on composition. If the zero strain assumption for the plane of lamellar interfaces is dropped, the coherent phase relations depend on the composition of the homogeneous precursor phase irrespective of whether the elastic constants show compositional dependence. In general, there is no single coherent solvus for a specific solid solution. The coherent solvus needs to be calculated individually for each possible composition of the homogeneous precursor phase instead. The analysis is by no means restricted to the exsolution of alkali feldspar, it can be applied to any coherent lamellar intergrowth.

## Conclusion

Equilibrium phase relations for coherent lamellar intergrowth of Na-rich and K-rich alkali feldspar from the exsolution of an initially homogeneous precursor alkali feldspar were calculated. The underlying Gibbs energy landscape was obtained by adding the elastic energy arising from the chemical segregation of ${\text{Na}}^+$ and ${\text{K}}^+$ in a coherent Al, Si–O tetrahedral framework to the Gibbs energy of strain-free alkali feldspar. The elastic problem was solved by minimising the elastic energy of the lamellar microstructure, which requires an iterative procedure. We find that the coherent equilibrium phase relations implicitly depend on the composition of the homogeneous precursor feldspar as well as on temperature. Accordingly, the critical temperature and the exact position and shape of the coherent solvus for alkali feldspar are different for homogeneous precursor feldspars with different compositions. Experimentally observed lamellar orientations are such that the total elastic energy of the lamellar microstructure is minimised. For the case at hand, the elastic constants were treated as independent of temperature and composition. If information on these dependencies should become available, they can be accounted for without fundamental changes to the analysis.

## References

[CR1] Abart R, Petrishcheva E, Kaessner S, Milke R (2009) Perthite microstructure in magmatic alkali feldspar with oscillatory zoning; Weinsberg Granite, Upper Austria. Mineral Petrol 97:251–263

[CR2] Abart R, Petrishcheva E, Wirth R, Rhede D (2009) Exsolution by spinodal decomposition II: perthite formation during slow cooling of anatexites from Ngoronghoro, Tanzania. Am J Sci 309:450–475. 10.2475/06.2009.02

[CR3] Angel R, Sochalski-Kolbus L, Tribaudino M (2012) Tilts and tetrahedra: the origin of the anisotropy of feldspars. Am Miner 97:765–778

[CR4] Bollmann W, Nissen H (1968) A study of optimal phase boundaries—case of exsolved alkali feldspars. Acta Crystallogr Sect A 24(5):546. 10.1107/S0567739468001178

[CR5] Brown WL, Parsons I (1984) Exsolution and coarsening mechanisms and kinetics in an ordered cryptoperthite series. Contrib Miner Petrol 86:3–18

[CR6] Cahn JW (1961) On spinodal decomposition. Acta Metall 9(9):795–801

[CR7] Cahn JW (1962) Coherent fluctuations and nucleation in isotropic solids. Acta Metall 10(10):907–913

[CR8] Connolly J (1990) Multivariable phase-diagrams—an algorithm based on generalized thermodynamics. Am J Sci 290(6):666–718. 10.2475/ajs.290.6.666

[CR9] Evangelakakis C, Kroll H, Voll G, Wenk H-R, Hu M, Kopcke J (1993) Low temperature coherent exsolution in alkali feldspar from high grade metamorphic rocks of Sri-Lanka. Contrib Miner Petrol 114:519–532

[CR10] Haussuehl S (1993) Thermoelastic properties of beryl, topaz, diaspore, sanidine and periclase. Z Kristallogr 204:67–76

[CR11] Holland TJB, Powell R (2011) An improved and extended internally consistent thermodynamic dataset for phases of petrological interest, involving a new equation of state for solids. J Metamorph Geol 29(3):333–383. 10.1111/j.1525-1314.2010.00923.x

[CR12] Hovis G, Delbove F, Bose M (1991) Gibbs energies and entropies of K-Na mixing for alkali feldspar from phase equilibrium data: implications for feldspar solvi and short range order. Am Miner 76:913–927

[CR13] Kroll H, Schmiemann I, von Cölln G (1986) Feldspar solid solutions. Am Miner 71:1–16

[CR14] Nye J (1957) Physical Properties of Crystals. Oxford at the Clarendon Press

[CR15] Parsons I, Fitz Gerald JD, Heizler MT, Heizler LL, Ivanic T, Lee MR (2013) Eight-phase alkali feldspars: low-temperature cryptoperthite, peristerite and multiple replacement reactions in the klokken intrusion. Contrib Miner Petrol 165(5):931–960. 10.1007/s00410-012-0842-5

[CR16] Parsons I, Fitz Gerald JD, Lee MR (2015) Routine characterization and interpretation of complex alkali feldspar intergrowths. Am Miner 100(5–6):1277–1303. 10.2138/am-2015-5094

[CR17] Petrishcheva E, Abart R (2009) Exsolution by spinodal decomposition I: evolution equation for binary mineral solutions with anisotropic interfacial energy. Am J Sci 309:431–449

[CR18] Petrishcheva E, Abart R (2012) Exsolution by spinodal decomposition in multicomponent mineral solutions. Acta Mater 60:5481–5493 23888123 10.1016/j.actamat.2012.07.006 PMC3719097

[CR19] Petrishcheva E, Tiede L, Schweinar K, Habler G, Li C, Gault B, Abart R (2020) Spinodal decomposition in alkali feldspar studied by atom probe tomography. Phys Chem Miner 47(7), JUN 7. ISSN 0342-1791. 10.1007/s00269-020-01097-4 10.1007/s00269-020-01097-4 PMC7319307 32624637

[CR20] Porter D, Easterling K, Sherif M (2021) Phase transformations in metals and alloys, 4th edn. CRC Press

[CR21] Ribbe P (1983) Chemistry, structure and nomenclature of feldspars, pages 1–19. Mineralogical Society of America

[CR22] Robin P (1974) Stress and strain in cryptoperthite lamellae and coherent solvus of alkali feldspars. Am Miner 59(11–1):1299–1318

[CR23] Smith JV, Brown WL (1988) Feldspar minerals, 1 crystal structures, physical, chemical and microtextural properties, 2nd edn. Springer-Verlag, Berlin

[CR24] Strang G (2005) Linear algebra and its applications, 4th edn. Brooks/Cole

[CR25] Tullis J, Yund R (1979) Calculation of coherent solvi for alkali feldspar, iron-free clinopyroxene, nepheline-kalsilite, and hematite-ilmenite. Am Miner 64(9–10):1063–1074

[CR26] Waeselmann N, Brown JM, Angel RJ, Ross N, Zhao J, Kaminsky W (2016) The elastic tensor of monoclinic alkali feldspars. Am Miner 101(5):1228–1231

[CR27] Willaime C, Brown W (1974) Coherent elastic model for determination of orientation of exsolution boundaries—application to feldspars. Acta Crystallogr A-Found Adv A 30:316–331. 10.1107/S0567739474010783

[CR28] Yund RRA, Tullis J (1983) Strained cell parameters for coherent lamellae in alkali feldspars and iron-free pyroxenes. Neues Jahrbuch für Mineralogie - Monatshefte 1983(1):22–34

